# Supplementary material for: Molecular and Cellular Characterization of the TH Pathway in the Sea Urchin Strongylocentrotus purpuratus
Source: Cells. 2023 Jan 10;12(2):272. doi: 10.3390/cells12020272 (PMC9856734; doi:10.3390/cells12020272)
Supplement: Supplementary file 1 [file cells-12-00272-s001.zip › cells-1741494-supplementary.pdf]

cells

MDPI

Article

Molecular and cellular characterization of the TH pathway in the sea urchin *Strongylocentrotus purpuratus*

Maria Cocurullo[1,#], Periklis Paganos[1#], Natalie J. Wood[2#] , Maria I. Arnone[*,1] and Paola Oliveri [2,*]

[1]Department of Biology and Evolution of Marine Organisms, Stazione Zoologica Anton Dohrn, Villa Comunale, 80121, Naples, Italy; maria.cocurullo@szn.it, periklis.paganos@szn.it, miarnone@szn.it.

[2]Centre for Life's Origins and Evolution; Research department of Genetics, Evolution and Environment, University college London, London, WC1E 6BT, UK; natalie.wood.15@ucl.ac.uk, p.oliveri@ucl.ac.uk.

*Correspondence: p.oliveri@ucl.ac.uk, mia.arnone@szn.it
#These authors contributed equally

Supplementary material

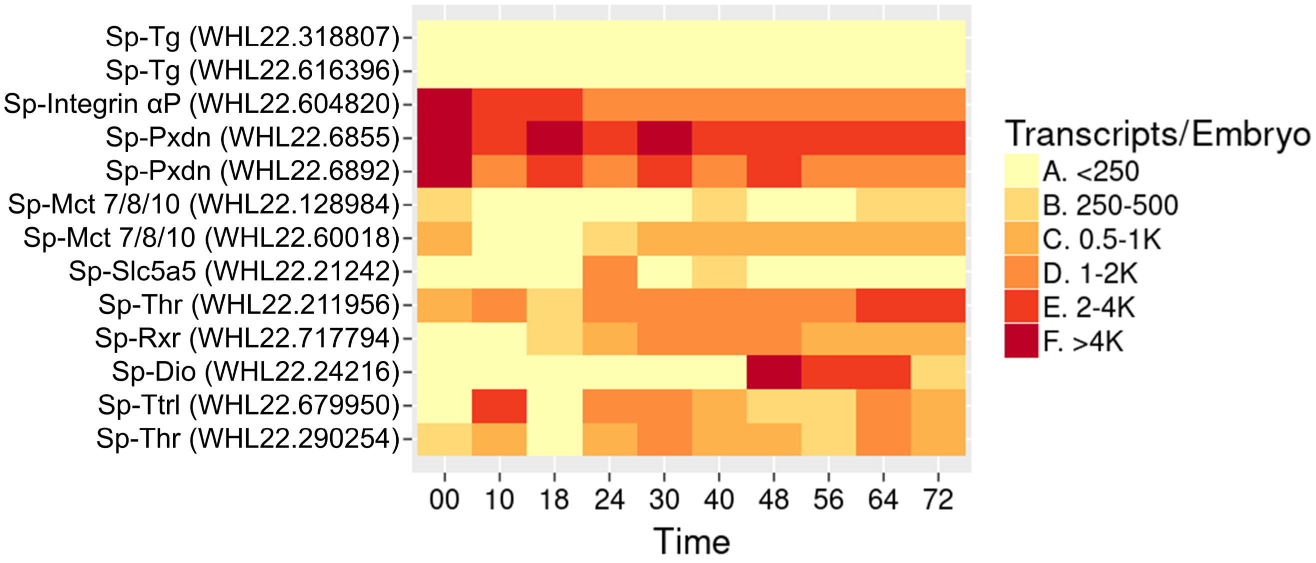

Figure S1: Expression profiles of putative sea urchin TH pathway components during early development. Heatmap was generated using the Quantitative Developmental Transcriptomes of *S. purpuratus* plotting tool (http://legacy.echinobase.org/shiny/quantdev/).

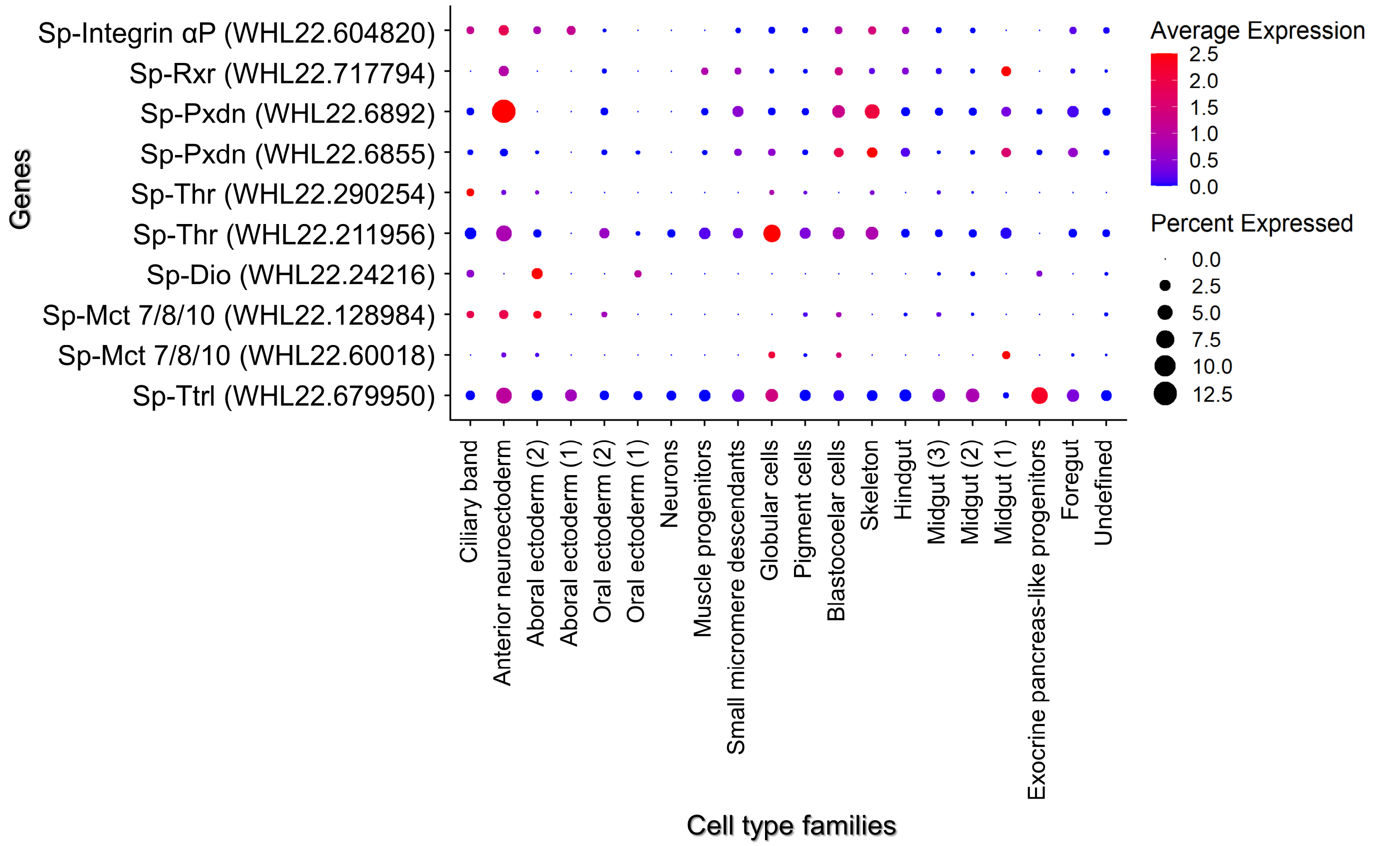

Figure S2: **Expression patterns of putative sea urchin TH pathway components at 2 dpf.** Dotplot showing the average expression of the TH pathway related genes present in our single cell libraries.

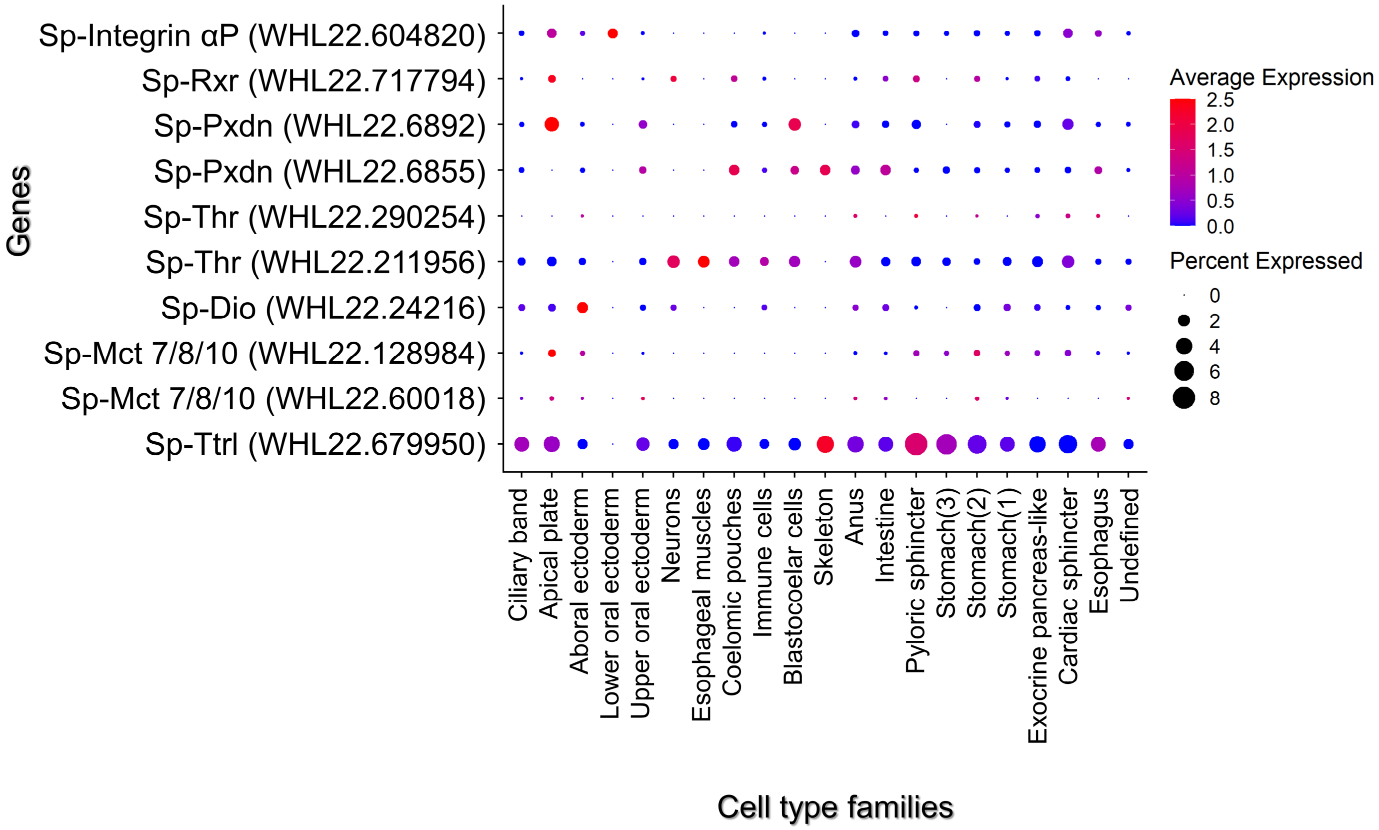

Figure S3: **Expression patterns of putative sea urchin TH pathway components at 3 dpf**. Dotplot showing the average expression of the TH pathway related genes present in our single cell libraries.

Table S1. Primers used to clone the gene of interests

Gene name	Primer sequence	
Sp-ThR	Sp-ThrF	GCCCTGAACCGTGTGTAGTA
	Sp-ThrR	ATTTAGGTGACACTATAGAGGAATCTCGTTTGGTCGGT
Sp-Pxdn	Sp-PxdnF	TGCGCTAGGAGAGGTCAAAA
	Sp-PxdnR	ATTTAGGTGACACTATAGGGGTGCTGAAGATTGTTGCA
Sp-DIO	Sp-DIOF	AGGAACTCGAGACACACTTCT
	Sp-DIOR	ATTTAGGTGACACTATAGTTCGTCGGTCATCAGCTGTT
